# Cell therapy approaches to autism: a review of clinical trial data

**DOI:** 10.1186/s13229-020-00348-z

**Published:** 2020-05-24

**Authors:** Jack Price

**Affiliations:** Institute for Psychiatry, Psychology, & Neuroscience, King’s College London, De Crespigny Park, Denmark Hill, London, SE5 8AF UK

**Keywords:** Autism, Cell therapy, Clinical trials, Stem cells

## Abstract

A number of clinical trials of cell therapies for autism spectrum disorder have been conducted, and some have published their outcomes. This review considers the data that have emerged from this small set of published trials, evaluates their success, and proposes further steps that could be taken if this field of endeavour is to be pursued further. A number of reservations arise from this tranche of studies, specifically the absence of identified therapeutic targets, and deficiencies in the therapeutic approach that is being employed. If this therapeutic direction is to be pursued further, then additional pre-clinical studies are recommended that might lead to improvements in patient stratification, biomarkers, the defined mode of action, and the preparation and identification of the therapeutic cells themselves.

## Background

Stem cell therapies are increasingly becoming applied to human patients. Since there are few cell therapies approved under any jurisdiction, most of these therapies are unlicensed. Some are undergoing clinical trials within conventional regulatory scrutiny, but the greater number are ‘direct-to-consumer’ products, seeking to bypass conventional regulation. In recent years, autism spectrum disorder (ASD) has joined the list of disorders for which such therapies are deemed by some to be suitable. Again, most of these are direct-to-consumer, but a number of clinical trials have commenced. A smaller number have now reached completion and have reported data. The purpose of this review is to consider the status of these clinical trials of cell therapies for autism, evaluate progress, ask whether it justifies the continuation of this approach, and if so, what steps need to be taken by clinical and preclinical researchers alike to improve the prospects of success.

### Clinical trials

What is the current status of clinical trials for ASD? In order to get an overview, I searched the US NIH Clinical Trials database (*clinicaltrials.gov*) using the search terms, Autism Spectrum Disorder AND Cell Therapy (searched December 2019). This revealed 37 items. Of these, just 14 were actual trials of cell therapies, and of these 13 were for ASD, one actually being a study of cerebral palsy (Table 1). This may or may not capture the complete picture worldwide. While registration of trials on this NIH database is only compulsory for NIH-supported studies, it does attract *bona fide* submissions more broadly. Nonetheless, it cannot be assumed to capture all trials of relevance. Of note, none of the 13 were based in Europe, and a comparable search of the European Medicines Agency’s Clinical trials register (*clinicaltrialsregister.eu*) revealed 58 registered trials for ASD, but none involving cell therapies (data not shown).

**Table 1:** Clinical trials for ASD logged onto *clinicaltrials.gov*

Status	Study title	Study identifier	Autologous/allogeneic	Cell type	Route of admin (where stated)	Reference
Withdrawn	Stem cell therapy in autism spectrum disorders	NCT01974973	Autologous	Bone marrow		
Completed	Safety and efficacy of stem cell therapy in patients with autism	NCT01343511	Allogeneic	Cord blood	Intravenous and intrathecal	2
Completed	Autologous bone marrow stem cell therapy for autism	NCT02627131	Autologous	Bone marrow	Intrathecal	4
Unknown	A clinical trial to study the safety and efficacy of bone marrow-derived autologous cells for the treatment of autism	NCT01836562	Autologous	Bone marrow	Intrathecal	
Completed	Autologous bone marrow stem cell therapy combined with psychological therapy and rehabilitation for autism	NCT03225651	Autologous	Bone marrow	Intrathecal	
Completed	Cord blood infusion for children with autism spectrum disorder	NCT02847182	Both	Cord blood	Intravenous	
Completed	Allogeneic umbilical cord mesenchymal stem cell therapy for autism	NCT02192749	Allogeneic	MSCs—umbilical cord	Intravenous	3
Withdrawn	Adipose-derived stem cell therapy for autism	NCT01502488	Allogeneic	MSCs—fat cells	Intravenous	
Completed	hCT-MSCs for children with autism spectrum disorder (ASD)	NCT03099239			Intravenous	5
Recruiting	Alzheimer's autism and cognitive impairment stem cell treatment study (ACIST)	NCT03724136	Autologous	Bone marrow		
Recruiting	Safety and efficacy of the transfusion of UCB in patients with an ASD depending on the degree of HLA compatibility	NCT03724136	Allogeneic	Cord blood		
Enrolling by invitation	Allogenic cord blood transfusion in patients with autism	NCT03786744	Allogeneic	Cord blood		
Unknown	Autologous bone marrow stem cells for children with autism spectrum disorders (autism)	NCT01740869	Autologous	Bone marrow	Intrathecal	
Recruiting	hCT-MSC in children with autism spectrum disorder (IMPACT)	NCT04089579	Allogeneic	MSCs—umbilical cord	Intravenous	
Completed	Autologous cord blood stem cells for autism	NCT01638819	Autologous	Cord blood	Intravenous	1

Of the 13 ASD/cell therapy trials, two had been ‘withdrawn’ and a further two were of ‘unknown status’. Seven were completed and four were active, either ‘recruiting’ or ‘enrolling by invitation’. Of the completed trials, just one had reported data on *clinicaltrials.gov*, but five could be traced to publications in scientific journals, and could, therefore, be subjected to analysis to ask: what scientific hypotheses underpinned these trials; what preclinical data supported the hypotheses; what clinical parameters governed the conduct of the trials; and finally, do the outcomes support the original hypotheses and provide a basis for a positive risk/benefit analysis that could justify further trials?

Of the seven, only one was a placebo-controlled trial [1], although a second study had a non-randomised control arm designated as ‘parallel assignment’ [2]. The remainder were Ph I/II open-labelled trials. For each, the primary rationale for the therapy was that ASD involves immune dysregulation and stem cell therapies can rescue such dysfunction. In most papers, however, this is not explicitly stated, and other therapeutic targets were also mentioned. For example, Lv et al. (2013) argue that a ‘combination of therapy modalities’ might be elicited by stem cell therapy, which appears to include ‘improving local blood perfusion to damaged areas through angiogenesis’ [2]. Sharma et al. target ‘brain hypoperfusion and immune dysfunction’. In none of the studies is a specific molecular target identified, although Riordan et al. do identify specific biomarkers [3] (see below).

The stem cells of choice and mode of administration are varied. They include human allogeneic cord blood mononuclear cells (CBMCs) and human umbilical cord mesenchymal stem cells (MSCs) in combination [2], autologous bone marrow mononuclear cells (BMMCs )[4], autologous CBMCs [1, 5], and MSCs alone [3]. Modes of administration were typically intra-venous (iv) infusion, though Lv et al. administered via two routes—iv for CBMCs and intra-thecally for MSCs. Sharma et al. use the intra-thecal route exclusively [4]. The injection regimens and follow-up periods varied considerably, as might be expected for such early-stage studies, from single-dose with 6-month assessment [5] through to four treatments over 9 months with follow-up over 21 months [3]. In each case, however, the dosing regime seemed arbitrarily fixed, and the basis for the choice was not indicated.

Considering the trial outcomes, the study by Chez et al. (2018) demands the most attention since it employs a placebo-controlled, cross-over structure [1]. Twenty-nine children between the ages of 2.4 and 6.8 years were given single iv injections of either autologous CBMCs or placebo. They were subjected to a comprehensive series of behavioural tests at 12 weeks—primarily vocabulary tests, plus cognitive, socialization, and communication assessments as secondary—then at 24 weeks, each was given the reciprocal treatment (CBMCs or placebo) then tested again after a further 12 weeks. The authors report no significant change in any test over pre-treatment assessment. In fact, outcomes on all behavioural parameters remain largely unchanged across the entire 49 weeks of the study.

The authors contrast this outcome with that of the study by Dawson et al. (2017), an open-label study on twenty-five children of similar age, again with autologous CBMC therapy, and a similar behavioural testing regime over 6 and 12 months. That study reported significant improvements across a range of parent-reported and clinician assessments covering socialisation, communication, and adaptive behaviours. They also reported improvements in eye tracking. The significant effects were visible at 6 months and remained stable over the 12 months of the study.

The obvious difference between the two studies is the placebo-controlled versus open-label structures, but it is also noteworthy that—as the authors themselves indicate—the improvement seen in the Dawson study is in line with that reported in control patients in a similar-aged Swedish cohort [6], and thus might be expected from the natural history of the disorder. The conclusion that emerges is that there is little support from these two relatively large, well-constructed studies to support this therapeutic direction for ASD. Autologous cord blood CD34+ cells seem not to have efficacy, at least over this time course and with this dosing regime. Nonetheless, two further studies (NCT02847182 and NCT04089579) appear to be in progress from this group of researchers.

The study by Lv et al. (2013) is similarly scaled, but more complex in structure. It involves two potential therapies: allogeneic CBMCs administered iv, or a combination of iv CBMCs together with intra-thecal administration of MSCs. Patients are boys and girls between 3 and 12 years of age. The study was spread across two centres, with one centre providing both the treatment arms, while the second centre provided the control group. All trial participants received behavioural therapy. The authors report significant improvements in all three groups in a range of behavioural outcomes—Childhood Autism Rating Scale (CARS); Severity of Illness of Clinical Global Impression, and Aberrant Behaviour Checklist—at 24 weeks following treatment. Most marked was the impact of the combined therapy particularly on the CARS scale where there was a 37.9% improvement.

The unconventional structure of this trial makes the analysis somewhat complex. The ‘control’ group was, in fact, a different study cohort in a separate centre, undergoing behavioural therapy under the guidance presumably of a separate group of clinicians. This is therefore not a randomly assigned control, and the authors do not report any steps to identify and isolate uncontrolled variables between the control and experimental groups. Since the two experimental groups were randomised, they are more easily compared directly. The combination group appears to do better than the CBMC group, but the report does not examine this comparison statistically.

Sharma et al. (2013) report an open-label study of a cohort of patients that differs significantly from those reported above by including adults. The age range varied from 3 to 33 years. It is also the most invasive. Patients are injected with GCSF, 1 to 2 days prior to treatment. Then, bone marrow cells are surgically removed from the patient via the iliac crest. Following isolation of CD34+ cells by FACS, this autologous BMMC cell preparation is injected intra-thecally. Follow-up is at irregular intervals from between 5 and 26 months. In addition, patients are subjected to positron emission tomography-computed tomography (PET-CT) following the injection of [^18^Fl] Fluorodeoxyglucose.

Since there is no control group, patients are assessed against pre-treatment behavioural assessments, and the authors report remarkable outcomes: 91% of patients showed behavioural improvements. But since any positive change is included however small, the proportion of patients that achieved significant improvement cannot be determined.

Since the patients are subjected to four distinct clinical interventions—GCSF injection, bone marrow aspiration, intra-thecal injection, and PET-CT—the risk benefit analysis in this study is important. The authors conclude that the procedure is ‘easy and safe’, and report only minor concerns with acute adverse events. Nonetheless, 3 patients (9%) suffered de novo seizures, and other ‘minor’ complications included spinal headaches, vomiting, and pain, either at the site of aspiration or injection. Long-term adverse events were not recorded. One notes that intra-thecal injection has a well-established risk [7] and that under-reporting of adverse events in regenerative medicine is a recognised issue [8]. An important question, therefore, is whether the risk-benefit profile for this approach makes it unethical. Certainly, it would seem to step outside of the guidance from the International Society for Stem Cell Research (ISSCR), which recommends that:

*Before launching high-risk trials or studies with many components, researchers should establish the safety and optimality of other intervention components, like devices or co-interventions such as surgeries*^1^.

There is no evidence presented to suggest that the extensive set of components in this study have been evaluated in this cohort of patients, either alone or in combination. Specifically, no risk-benefit analysis is presented for this complex therapeutic approach.

The final study reported in this *clinicaltrials.com* search is from Riordan et al. (2019) [3]. The question immediately arises as to whether this should be considered a genuine clinical trial, or rather presents an example of the ‘pay-to-participate’ studies that have been shown to use *clinicaltrials.gov* as an advertising vehicle for unlicensed therapies [10]. Reports suggest that this is indeed the case^2^, and the authors themselves declare their financial conflict of interest in the publication.

The study itself is an open-label trial of unmatched, allogeneic, bone marrow-derived MSCs in 20 ASD children aged between 6 and 16, all but one boys. Patients were given four treatments over a total of 37 weeks. Safety endpoints were assessed by clinicians at six time points through the study, and efficacy endpoints in the form of parent assessed behavioural outcomes were assessed at five time points, following a pre-study assessment. The study reports few adverse events and none that were serious. Five patients, however, did not complete the study, and adverse events in those patients were not reported.

In relation to efficacy, the study claims statistically significant outcomes in both behavioural assays employed—CARS and ATEC (autism treatment evaluation checklist). What is striking about the primary data, however, is how variable the outcomes were at each time point, and how flat the progression curve is. The improvement the authors claim is not immediately visible in these analyses. The study also reports individually the data on eight patients that showed significant clinical improvement, but not on the remainder, who presumably did not improve.

Notably, this study, unlike the others considered here, measures two serum cytokines (MDC and TARC) to evaluate the impact of therapy of these biomarkers of inflammation. The authors claim statistical improvement in these measures also, but again the primary data appear too variable and flat to support this contention.

### Reservations

These studies present a mixed picture. The only placebo-controlled study resulted in a negative outcome, while the open-labelled studies provided mixed and, in most cases ambiguous, outcomes. Before considering where such studies might go next, some reservations need to be voiced regarding the routes that have been undertaken so far. Two areas present particular concerns.

#### Therapeutic target

None of the studies reviewed here have a firm scientific basis. As we have seen, most invoke ‘immune dysfunction’ as a component of ASD pathology, and thereby justify the cell therapy approach on the basis that the various cell types proposed have ‘immuno-regulatory properties’. This argument is weak. The authors of each paper cite the extensive data that support the ‘immune dysfunction’ hypothesis. These studies are extensive and have been reviewed at length in several recent publications [11–14]. Briefly, the supporting data fall into three categories. First, there are epidemiological data, supported by animal experimentation, that suggest that exposure to inflammatory stimuli during pregnancy leads to an increased likelihood of a postnatal diagnosis of ASD [15]. A widely proposed mechanism is exposed in utero to pro-inflammatory cytokines, such as Il-1beta, Il-6, and interferon-gamma. The second body of data reports clinical studies showing altered levels of cytokines and/or immune cell populations in autistic individuals themselves [16, 17]. Third, there is genetic data suggesting an association between ASD and some genetic loci, known to be involved with immune function [18]. An example would be the association of particular MHC alleles with autism [19]. These various arguments seem sound and do indeed implicate the immune system in ASD etiology. Nonetheless, to put this in context, equally large volumes of research on ASD point in different directions, a synaptic pathology [20], for example, or the reported association for ASD with other neurotoxic events [21] or hormone imbalances [22]. While these alternative patho-physiological pathways are not necessarily mutually incompatible, the best that can be said currently is that the data on the pathophysiology of autism points simultaneously in multiple directions, that multiple routes exist into ASD, and that a diagnosis of ASD crosses multiple sub-populations of patients [23].

Nonetheless, even accepting the ‘immune dysfunction’ data at face value provides inadequate support for these clinical interventions. The data actually address two distinct categories of hypotheses. The genetic, epidemiological, and animal data support the hypothesis that inflammation—and/or the response to pro-inflammatory stimuli—contributes to ASD etiology in utero. On the other hand, the clinical data suggest ASD patients themselves have disturbed immune function. These are distinct hypotheses, which may or may not be related. Many of us endure disturbed immune dysfunction because of bacterial or viral infections, stress, or myriad other effectors that impact immune function, yet we do not have autism. There is no suggestion that such immune activation in the adult is associated with adverse developmental events. Similarly, many mothers suffer viral infections during pregnancy yet give birth to neurotypical children. The authors of these studies present no evidence to suggest that these two parameters are associated with ASD. More significantly, they do not address the question of which of these two risk factors—the developmental and the acute—they are seeking to impact, or pivotally, what the acute sequelae of these factors are that the therapy seeks to address. The exception here is the study by Riordan et al. (2019) where a clear case is made for the involvement of plasma cytokines [3]. This has the virtue of having a clear biomarker for the impact of the therapy on the proposed mode of action.

Reversing the developmental disturbance would seem forlorn. There is no robust way to identify the subset of patients (probably quite small) whose ASD is the result of an immune disturbance in utero, and even were the cohort identifiable, why should acute treatment with immune-regulatory cells reverse this long-standing dysfunction? There is little data on the nature of the immunological memory that must underpin this pathophysiology, but it is surely epigenetic in nature. If the patient’s immune cells carry an epigenetic signature that is somehow associated with the autism phenotype, how will the engraftment of more of the patient’s CD34+ stem cells—presumably carrying the same epigenetic signature—rectify anything?

If the target of the therapy is to reverse the acute immunological imbalance, then that becomes a credible objective, but leads to two further reservations, patient selection and the specific therapeutic approach, addressed below. But to conclude this point, it is surely inadequate to cite ‘immune dysfunction’ as the therapeutic target for these studies. What is the specific dysfunction that is being proposed, where is the evidence that that dysfunction is expressed in a particular cohort of patients, and what is the anticipated mechanism by which the cell therapy seeks to rebalance that dysfunction?

#### Therapeutic approach

Whether or not a credible case can be made for an immunological approach to the treatment of ASD, there seems to be little justification for a strategy involving the iv injection of CD34+, even less for an intra-thecal injection, which given its invasive nature and the absence of pre-clinical support for its use in this indication would contravene the ISSCR guidelines. The CD34+ stem cell population, isolated from either bone marrow or cord blood, has a long history as a therapy for a range of haematological disorders [24]. Efficacy in these instances relies primarily on the stem cell properties of the CD34+ cells, specifically, the potential to generate blood cells. More recently, this approach has been adopted for other conditions, for which there is evidence for an immune component, an example of relevance to this discussion being multiple sclerosis [25].

Two manipulations almost invariably accompany haematological stem cell therapy. First, the patient typically undergoes a ‘conditioning regimen’ in order to ablate the host immune cells. This removes malfunctioning cells, as in the case of leukemias, and generates an empty niche for the engrafted cells to occupy. Second, the CD34+ cells are mobilised by the injection of G-CSF (granulocyte-colony stimulating factor). This acts to increase the circulating concentration of the hematopoietic stem cells by reducing SDF-1 (stromal cell-derived factor 1) activity, thereby releasing CD34+ cells from their niche in the bone marrow [26]. In none of the studies reviewed here is the first of these steps undertaken, presumably because this would constitute an intolerable risk for the patients. Yet, the failure to ablate undermines the therapeutic strategy: when CD34+ cells are injected into the patients iv, there is no cell compartment prepared into which they can move. How the cells are expected to behave in this circumstance is not explained, and none of the studies cite biodistribution experiments that would show whether the cells survive and where they actually go in the body, but it seems likely that a few cells will home to the bone marrow, and the rest will be removed.

G-CSF mobilisation is performed in the Sharma et al. study even though the CD34+ cells are harvested by bone marrow aspiration [4]. Why patients would be treated to mobilise cells from the bone marrow into the circulation, if cells are subsequently to be harvested from the bone marrow is not explained.

The studies in which cells are injected intra-thecally makes even less logical sense. First, intra-thecal injection is considerably more invasive than iv injection. It is a serious surgical intervention that risks damaging neural tissue and has a range of well-documented complications [7]. It is conventionally used in two circumstances, first to administer pain relief in situations of severe pain. Second, it is the route of administration for some cytotoxic drugs during cancer therapy [27]. There is no precedent, as far as I am aware, for the injection of bone marrow stem cells via the intra-thecal route, and none for its use in this indication. Sharma et al. justify their intra-thecal route on the basis that it: ‘enhances the possibility of the maximal number of transplanted cells “homing” onto damaged sites.’ They do not, however, say what these damaged sites are, or cite any data to suggest that there is indeed damage. Again, there are no biodistribution data, so whether the cells ‘home’ to sites of damage, or anywhere else, is not documented. Again, this is not consistent with ISSCR guidelines, which recommend:

*‘Careful studies of biodistribution, assisted by ever more sensitive techniques for imaging and monitoring of homing, retention and subsequent migration of transplanted cell populations is imperative for interpreting both efficacy and adverse events’.*

Both these groups argue that intra-thecal injection is safe. Lv et al. suggest the injections were ‘well tolerated without immediate longterm side effects’, and believe that there is an acceptable risk/benefit ratio. Three of 32 patients in the Sharma et al. study suffered seizures. In addition, among the adverse outcomes were spinal headache, nausea, vomiting, and pain. Nonetheless, these authors consider the procedure safe. Such a sanguine approach seems difficult to justify. Complications with intra-thecal administration are well-documented, including damage to the spinal cord or cauda equina [7]. Moreover, the US FDA (Food and Drug Administration) currently only approve its use for three medications—morphine, ziconotide, and baclofen—in severe pain, or life-threatening indications such as cancer [27]. Continuing this approach without a clearer justification for this mode of administration seems unwarranted.

### Next Goals

In light of these reservations, how might further pre-clinical work improve the prospects for a successful cell therapy approach to ASD? There are five clear areas where progress is needed.

#### Mode-of-action

The proposal that ASD is the result of ‘immune dysfunction’ is inadequate. First, the evidence that inflammation plays a role in the pre-natal pathology of the disorder is not a strong basis for an immunological intervention, unless a residual immunological imbalance can be identified. There is indeed evidence for acute imbalances in immune regulators in ASD, such as those plasma cytokines cited by Riordan et al. (2019) [3]. Various other reports suggest a reduction in regulatory cytokines such as IL-1ß, IL-6, and IL-8 in ASD patients [17], and a reduction in regulatory T cells [28]. Yet more reports suggest an increase in cytokines with immunosuppressive roles, such as IL-35 (Ref [29]). These are all potential therapeutic targets for strategies to rectify the ‘immune dysfunction’ associated with ASD. If the cell therapy approach to ASD is to be placed on a firm scientific basis then a link needs to be built between these mediators of immune dysfunction and the mode-of-action of the cell therapeutic. This would then facilitate the generation of potency assays for the cells themselves (see below), biomarkers for efficacy, and a real test of the immune dysfunction hypothesis: namely, if the dysfunction is reversed, does this bring about an improvement in the core symptoms of the disorder.

This last point is key: currently, when studies such as that of Chez et al. fail, we cannot say whether it was a failure to restore immune regulatory balance, or whether balance was regained, but had no impact on behaviour. The hypothesis is not actually being tested by the study.

#### Patient Stratification

While the studies cited here had inclusion and exclusion criteria, there was no systematic stratification of ASD patients (although some excluded those diagnosed with Asperger’s syndrome). Within a broad specification, all ASD patients were apparently accepted as candidates for therapy. In the extreme case, both adults and children were included [4] making interpretation of the outcomes extremely complex.

The first level of stratification that seems appropriate is to select patients who show evidence of immune dysfunction. Published data do indeed suggest that ASD is associated with acute immune dysfunction, as noted above, but those data also suggest that this represents only a sub-set of patients. Several authors have reported altered levels of immunomodulatory factors specifically in patients with a more regressive form of autism (see [17] and citations therein). Estimates of the proportion of ASD patients with this regressive form vary considerably depending on the precise definition used, but seem to constitute between 15 and 50% of the total ASD patient population [30]. This fits well the consensus in the field that ASD is a complex disorder, with a broad range of risk factors (genetic, infectious, gastro-intestinal, neurotoxic), an enormously variable progression, and a spectrum of co-morbidities. I doubt any autism clinician or researcher would support the view that immune dysfunction was the single primary cause of ASD, yet in none of the studies cited here (except one [3]) was there an attempt to identify a specific immune correlate of the disorder, and in none at all were patients selected with that demonstrable immune dysfunction. Hence, each trial was almost certainly treating a cohort of patients some of whom had immune dysfunction but most of whom did not. This lessened the power of each study substantially. It also begs the question of how to interpret open-label studies that report a high success rate. While reported as a success, such results actually undermine the hypothesis that cell therapy is acting by restoring immune imbalance, since most of those patients would not have had a demonstrable immune imbalance. Either these studies selected an atypical ASD cohort or the rate of success has been overestimated—entirely possible in open-label trials—or this is not the mode of action of the therapy. As a minimum going forward, uncontrolled variables, such as rate of progression, need to be monitored and incorporated into the data analysis.

The third reason to engage with patient stratification is the risk/benefit assessment. All these studies claim to show that their therapy is safe, and certainly, major adverse events were broadly absent. Nonetheless, as noted above, the intra-thecal route particularly has demonstrable risks. The risk-benefit analysis will alter, therefore, depending on the quality of life and prognosis for different ASD sub-groups. As we well know, many ASD patients grow to be independent, well-balanced adults, with good quality of life, who do not consider themselves to be disabled in any sense. I would question whether cell therapy would ever be ethical for this group, who are not themselves competent to consent, and who might a priori be considered to have an unfavourable risk-benefit profile. The ISSCR guidelines suggest that where informed consent cannot be provided directly, then ‘*study procedures should be limited to no greater than a minor increase over minimal risk*’.

#### Biomarkers

Implicit in the foregoing discussion is the need and opportunity to employ biomarkers in trials of putative cell therapies in ASD. This is not a trivial undertaking and is perhaps the principle area where robust pre-clinical studies are required. The association noted above between ASD (particularly regressive ASD) and cytokine imbalance raises the possibility of using plasma cytokine levels as biomarkers for the effectiveness of cell therapy treatment. Such cytokines are currently used as biomarkers for some conditions, though their use has its difficulties [31]. One problem is that encountered in the Riordan et al. study, where the variance in plasma cytokine levels across the cohort was so great that average values become unhelpful. Nonetheless, cytokine surveillance may be a step towards robust biomarkers to measure the impact of cell therapies aimed at immune dysfunction. Such biomarkers are unlikely to ever become surrogate markers of efficacy, but that is not the issue in this instance. Behavioural tests for the core symptoms of ASD are well-established, and surrogates are not required. What is required, however, is a means to test the primary hypothesis: if immune dysfunction in specifically targeted patients is reversed, does this impact the cardinal symptoms of ASD? This can only be addressed, as noted above, if biomarkers are in place to measure the impact of the therapy on immune dysfunction.

Some work to identify biochemical markers has begun. The team associated with the Lv et al. study have subsequently reported increases in NGF in the CSF of patients treated with intrathecal and IV cell therapy [32]. Unfortunately, in such hypothesis-free studies, such a change might be a biomarker of efficacy, but just as probably, the change is a damage response to the cells, or to the intra-thecal injection itself.

#### The cell therapeutic

Finally, none of these studies give enough attention to the cells themselves. There are no release criteria for the cell preparations, except the most perfunctory (e.g. cell viability). There are no potency assays. For many cell therapies, potency assays are challenging because the mode-of-action of the cells is genuinely unknown. In the studies considered here, where restoring immune dysfunction is the proposed mode-of-action, devising appropriate potency assays could have been relatively easily implemented. Whether any of the patients in this study received cells that were actually immune-modulatory is unknown, but could have been tested. This is particularly relevant to the MSCs in, for example, the Riordan trial, since they are known to vary enormously in their therapeutic potential between preparations. Just freezing then recovering cells is known to impact the immune-modulatory activity of the cells (see [33] for review of this issue). This study uses the cell surface markers and the tri-lineage potential of the MSCs in place of potency assays. But though this potential defines MSCs, it has no relevance to the immune-modulatory activity of the cells, which is the putative therapeutic property.

## Conclusion

The published trials considered here are small in number and scale and permit therefore only a preliminary assessment of the potential of cell therapies for the treatment. The studies themselves vary in terms of the patient cohorts treated, the cell therapy of choice, the time course of the study, and the dosing regime. This makes them difficult to compare, and makes generalisations hard to derive. Nonetheless, this review has proposed a number of developments that would improve the validity and likelihood of success of future endeavours in this field. Whether any such improvements have been incorporated into the further studies now in progress (Table 1) remains to be seen.

One final comment seems appropriate. Since all the studies claim to show that their methodology is safe, further uncontrolled studies seem difficult to justify. The purpose of open-labelled phase I/II trials is to demonstrate safety. If that is achieved, then further such studies are redundant, and thereby unethical. Clearly, the only way we will know if cell therapies can have an impact on ASD is via properly placebo-controlled studies. This is disputed by some but remains the majority position among regulators and clinical scientists themselves [34]. Roughly 90% of drugs fail in clinical trials, and most fail for efficacy or safety reasons [35]. The data on advanced therapies is currently too sparse to analyse robustly, but the experimental nature of these therapies means that their success rate is unlikely to be higher. This means that the overwhelming majority of patients taking part in trials such as those considered here are receiving treatments that are unsafe, ineffective, or both. Parents and clinicians would do well to remember that these patients, for the most part, are children, unable themselves to give consent. In many cases, the future quality of life is very difficult to assess. How legitimate is it to expose these individuals to risk with such a low probability of success?

## Data Availability

Not applicable.

## Abbreviations

ASD: Autism spectrum disorder
ATEC: Autism treatment evaluation checklist
BMMC: Bone marrow mononuclear cells
CARS: Childhood Autism Rating Scale
CBMCs: Cord blood mononuclear cells
GCSF: Granulocyte-colony stimulating factor
CSF: Cerebrospinal fluid
FACS: Fluorescence-activated cell sorting
IL-1ß: Interleukin 1 beta
Il-6: Interleukin 6
Il-8: Interleukin 8
Il-35: Interleukin 35
ISSC: International Society for Stem Cell Research
iv: Intravenous
MDC: Macrophage-derived cytokine
MSC: Mesenchymal stem cells
NGF: Nerve growth factor;
NIH: National Institutes of Health;
PET-CT: Positron emission tomography-computed tomography
SDF-1: Stromal cell-derived factor 1
TARC: Thymus and activation-regulated chemokine

## Footnotes

1 See Ref [9] for summary of ISSCR guidance.

2 https://www.the-scientist.com/news-opinion/experts-question-rationale-for-stem-cell-trial-for-autism-66226. Sourced 2/12/19
